# Structural response of mandibular first molars in the presence of proximal contacts: finite element analysis with antagonist teeth and alternative loading applications

**DOI:** 10.1007/s00784-025-06290-9

**Published:** 2025-03-28

**Authors:** Saúl Dorado, Jesús R. Jimenez-Octavio, Paula Riaza, Ove A. Peters, Ana Arias

**Affiliations:** 1 Department of Mechanical Engineering, Escuela Técnica Superior de Ingeniería ICAI, Universidad Pontificia Comillas, Madrid, Spain; 2 MOBIOS Lab, Institute for Research in Technology, Escuela Técnica Superior de Ingeniería ICAI, Universidad Pontificia Comillas, Madrid, Spain; 3 Department of Conservative and Prosthetic Dentistry, School of Dentistry, Complutense University, Plaza Ramon y Cajal s/n. Ciudad Universitaria, Madrid, 28040 Spain; 4 Oral Health Centre, School of Dentistry, The University of Queensland, Herston, QLD Australia

**Keywords:** Biomechanics, Dental tissue, Finite element analysis, Functional load, Stress distribution

## Abstract

**Objectives:**

To compare the mechanical responses of a mandibular molar under functional loads using antagonist teeth and different loading applications and configurations.

**Methods:**

A cone-beam computed tomography of a human mandible and maxilla was used to build 16 different three-dimensional models, including four mandibular configurations [single-tooth model (first mandibular molar-M), and inclusion of mesial (mM), distal (Md) or both proximal contacts (mMd)] and occlusal load applications either with antagonist teeth or alternative Finite Element (FE) models [point load (PL), distributed surface load (SL) and rigid metal sphere (MS)]. FE analysis was performed. Equivalent von Mises (VM) stress was calculated along the entire dentin and periodontal ligament of the first mandibular molar. Maximum VM stresses were compared among the different mandibular configurations and loading applications.

**Results:**

The highest and lowest VM stress at 50 and 100 N corresponded respectively to the single-tooth SL model (5.78 and 11.5 MPa) and to occlusal load application with antagonist teeth and proximal contacts (2.08 and 3.58 MPa). Maximum VM stresses were consistently located at the cervical area of the mesial root and decreased when adjacent teeth were present.

**Conclusions:**

Highest stresses are located in the cervical area of the mesial root of mandibular molars, but the biomechanical behavior depends on the presence of proximal contacts and the loading methodologies used. Single-tooth models represent the worst structural scenario.

**Clinical relevance:**

Incorporating antagonist teeth and proximal contacts into FE models enhances the biofidelity of dental biomechanics simulations, enabling more accurate extrapolation to clinical conditions.

## Introduction

One of the main reasons for tooth extraction is fracture, either in the form of complicated crown-root fractures of non-treated teeth or vertical root fractures of root canal-treated teeth [1]. Importantly, deeper understanding of the biomechanical response of the dental complex may help to explain failure mechanisms. In fact, various models from in vitro studies trying to simulate various conditions in controlled laboratory environments to computational studies have been developed to better understand dental biomechanics and failure mechanisms. Finite element (FE) analysis has been widely used in the last few decades to understand and predict biomechanical phenomena. However, FE practices in biomechanics continues to pose a challenge for model development [2] and the results derived from oversimplified experimental or computer-simulated models may not be representative of the natural behavior of teeth [3].

When building a biomechanical model to analyze the mechanical response of teeth, it is necessary to consider various boundary conditions [3]. Whether the studied biomechanical models are experimental or computer-simulated, achieving the highest possible degree of biofidelity should be the objective of a biomechanical study [3, 4].

During occlusion, maxillary and mandibular teeth come in contact exerting compressive (occlusal) loads on the structural tissues of teeth. These structural tissues are enamel, dentin, pulp, periodontal ligament (PDL) and bone (both trabecular and cortical) [3, 5–7]. Enamel is typically the first material to contact antagonist teeth in occlusion and described as a biological ceramic due to its crystalline structure, which resembles ceramic materials [8]. Its structure is composed of enamel rods perpendicular to the dentin-enamel-junction [9]. Dentin is the tissue subjacent to enamel, and represents the major volume of the tooth, serving both as the elastic support for enamel and the protection for the internal dental pulp [10]. Its structure is characterized by dentinal tubules aligned perpendicular to the dental pulp towards the dentin-enamel-junction or the exterior cementum [5]. PDL surrounds the external surface of the root that is covered with cementum. It is a fibrous tissue, and its fibers are attached to the alveolar bone in one end and to the root cementum in the other end. Similar to dentin and enamel, the alignment of these fibers is dependent on their place of attachment [6]. PDL fibers are attached to the bone that act simultaneously as the support of teeth [6] and the receiver of the transmitted loads from the PDL [6, 7].

Several authors have reported that physiological factors such as age [11, 12], gender [11, 13], loading rate [14, 15] or salivary lubrication [16] might change the loading conditions and the mechanical response of oral biological tissues. Further studies have reported on the mechanical effects of functional and parafunctional loads in magnitude [17, 18], trajectories [19, 20] and frequency [21]. A demographic analysis of vertical fractures indicated a higher prevalence in mandibular molars, females and older patients [22].

At the same time, FE analysis, a tool widely applied in biomechanical studies and dentistry, is useful for studying complex biomechanical systems that are difficult to directly study in vivo or in vitro. FE models allow the virtual reproduction of biological structures, providing information about the mechanical response of this structures under different simulated clinical conditions. Among other applications in the field of dentistry, FE analysis has been used to numerically describe the behavior of dental materials [23], implants [24], restoration techniques [23] and teeth [25].

However, so far, the information provided by the literature include mathematical simulations that tend to result in simplified biomechanical models [3]. A common simplification is the use of point loads or surface distributed loads to represent the occlusion of teeth [26]. As described before, while this simplification may provide results somewhat close to reality, it is yet to be studied the reliability of FE models. FE analysis could provide further information about the mechanical response of teeth using more complex biomimetic biomechanical models with antagonist teeth and simulating real clinical occlusal contacts. Moreover, the presence of adjacent teeth has not yet been considered. Most prior work has analyzed mechanical responses with a single tooth [27, 28] or implant [24]. While this information might be useful, the simulated biomechanical response of a single tooth analyzed with excessive simplification might not translate to a clinical situation accurately and could lead to an underestimation or overestimation of the mechanical results obtained.

Therefore, the aim of this computational study was to compare the mechanical response of a mandibular molar under a functional (biting) clinical load using antagonist teeth and three commonly used FE loading applications for comparison. A second objective was to validate the stress distribution in the different structural tissues when a single tooth model is used in comparison with FE models in which adjacent teeth are present.

## Materials and methods

### Study objective and criteria of interest

FE analysis was used to evaluate Equivalent von Mises (VM) stress distributions in mandibular molars under functional mastication forces in a model obtained from a real human mandible and compare it to different loading applications. The method of construction of the 3D models, the different mandible configurations and the load application methods used are described below, as well as the biomechanical model and its boundary conditions.

### Model construction

To create biofidelic FE models, a cone-beam computed tomography (CBCT) scanner of the mandible and maxilla from a 50-year-old adult female patient was used. The resolution of the small field of view (4 × 4 cm) CBCT scan was 75 μm (Carestream 8100 3D, Carestream Dental LLC, Atlanta, GA). The DICOM files were initially imported in an image software (VGStudio, Volume Graphics-Hexagon, Stockholm, Sweden) to generate stereolithography surfaces (STL) files from the scanned samples.

Segmented files for enamel, dentin and bone were created from the mandible scan and a non-segmented STL was created from the maxillary scan. Once converted into STL, they were imported onto Geomagic Studio (3D Systems, Rock Hill, SC, USA) to eliminate unwanted noise and sharp geometries from the models. In this software, the STL model of each segmented structure was smoothed with normal vectors of the surface oriented into the same direction to generate an IGES file containing a 3D volume. IGES files were then imported into SolidWorks 2022 (Dassault Systèmes, Paris, France) to build different models for simulation. Additionally, the PDL was modeled using the space left between the segmented dentin and the alveolar bone, having approximately 0.2 mm thickness throughout the entire geometry [29, 30]. Both mandibular first molars and their adjacent and antagonist teeth were extracted from the complete model for further study along with their surrounding cortical bone, while the trabecular bone was modeled as a solid that filled the inside of the cortical layer. The maxilla was only used for loading application and to model the contact between the occluding antagonist surfaces. Hence, only the coronal section, consisting of enamel was extracted from the scan and implemented into those final models where the maxillary teeth were present. No more maxillary tissues were included for ease of calculations, as stresses were evaluated on the mandibular first molar.

**Fig. 1:**
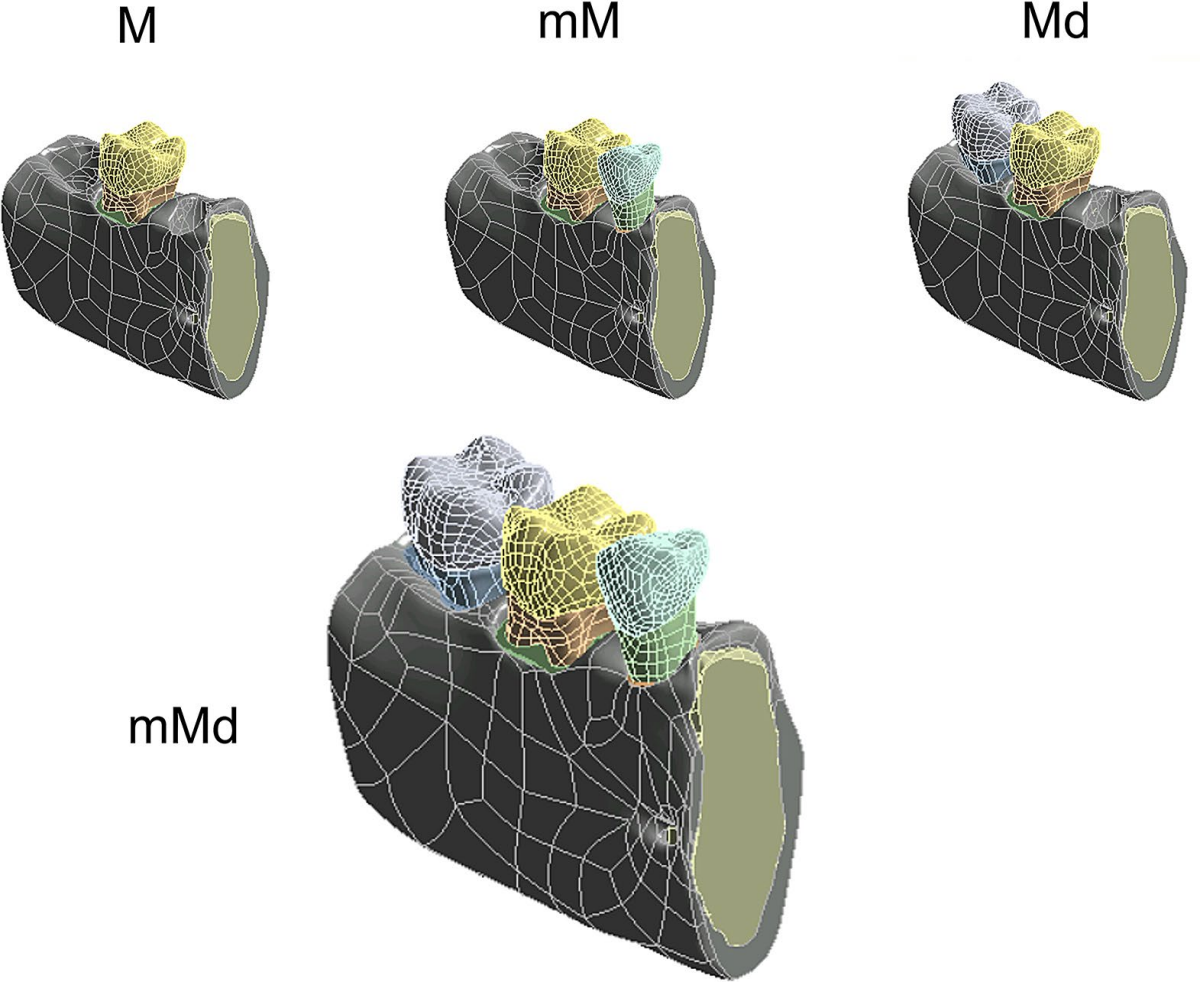
Three-dimensional models representing the four mandibular configurations studied: single-tooth model (M), both models with one proximal contact, mesial (mM) or distal (Md) and the mandibular model with both proximal contacts (mMd)

Four mandibular configurations were analyzed in this study. The first configuration was a single-tooth model including only the first molar (4.6) (M) and no adjacent teeth. The second and third configurations included the first molar and an adjacent tooth, either the second premolar (4.5) (mesial adjacent tooth model (mM)) or the second molar (4.7) (distal adjacent tooth model (Md)). The fourth configuration included both adjacent teeth, the first molar with both mesial and distal proximal contacts (mMd). Figure 1 shows the model with the four mandibular configurations.

The final model was then imported into ANSYS 22.1 (Ansys, Canonsburg, PA, USA), where it was discretized and simulated. A simple mesh convergence test was run to confirm that the simulation results were independent from further increases in mesh density. Seven different meshes were used for the purpose, the one used in the present study and 6 more with a difference in maximum mesh skewness up to 10% (3 with a 10–15% element increase and 3 with the same 10–15% element decrease). Maximum VM stress was used as the reference. A maximum of 3% relative variation was established as an acceptable threshold and fulfilled by all meshes.

The final generated meshes had between 300,000 (M) and 450,000 (mMd) elements with 380,000 and 730,000 nodes, respectively. The element type used was TET10. The mesh quality metric chosen was mesh skewness, which, for the mesh with the lowest quality (mMd), was found to be 0.26 ± 0.14.

### Material properties

Material properties in this study were selected to realistically represent the condition of the orofacial system of the subject of study (adult female with no caries or restorations), as these vary depending of age and gender, among other variables [3].

As shown in Table 1, each segmented tissue was defined according to a previous review [3] and all materials were considered to be isotropic linear elastic. Mechanical properties of enamel were determined with a linear interpolation with age from previous results [31]. Characteristics of the rest of the materials were determined by averaging the results of previous studies [32–36].

**Table 1:** Summary of the mechanical properties for the different tissues of the dental complex used in this study

Tissue	Elastic Modulus (GPa)	Poisson´s ratio
***Enamel***	85.4	0.303
***Dentin***	17.8	0.31
***PDL***	50e-3	0.45
***Cortical Bone***	18.3	0.3
***Trabecular Bone***	1.5	0.3

### Loadings and boundary conditions

Four different static structural loading application methods were used in all mandible configurations described before. Load direction, magnitude and application points were selected to realistically represent the baseline masticatory function of an adult female with no caries or restorations.

The first loading application method tried to simulate the clinical occlusal contacts of the patient. The occlusal masticatory load distribution of the patient was recorded with OccluSense^®^ electronic pressure sensor. The sensor allowed the recording of the masticatory forces in 256 pressure levels and registered them in the Occlusense iPad App (Bausch, Köln, Germany). The masticatory load distribution was transferred to the antagonist teeth (model A) with loading application between the 3D mandibular and maxillary models (teeth 1.5, 1.6 and 1.7) [37]. Occlusal surfaces were defined according to the masticatory load distribution, both arches were positioned in occlusal contact and load was applied in the enamel of the maxillary model.

In addition, three loading FE models previously used in different studies [27, 28, 38–40] were also applied to the four mandible configurations (M, mM, Md, mMd). These loading applications were:

FE model with distributed surface load application in occlusal contact areas (SL) [39]. The occlusal contacts were imprinted in the mandibular teeth and defined as distributed loading surfaces.

FE model with point load in the occlusal contact areas (PL) [27, 28]. The centroid of each occlusal contact surface was defined as loading points.

FE model with load application using a rigid metal sphere (MS) [38, 40]. The sphere was positioned over the center of mass of the enamel with a maximum penetration of 0.5 mm. The intersection volume was subtracted from the sphere to define surface contacts to transmit the load to the enamel.

**Fig. 2:**
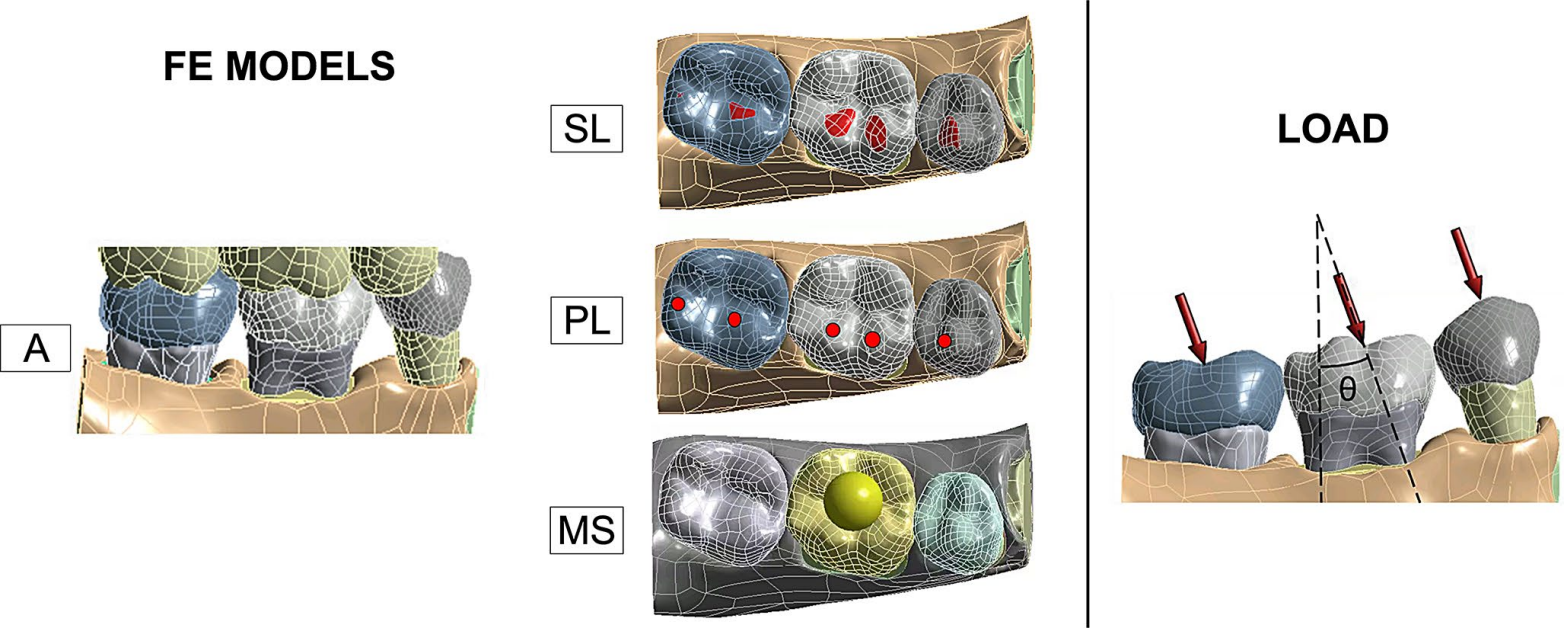
Representations of the mandibular configuration that included the first mandibular molar with both mesial and distal adjacent teeth (mMd) for the four different loading applications, along with a simple scheme on the loading direction applied (θ=17.5º)

Figure 2 shows the four different loading applications and loading direction in mMd configuration.

**Fig. 3:**
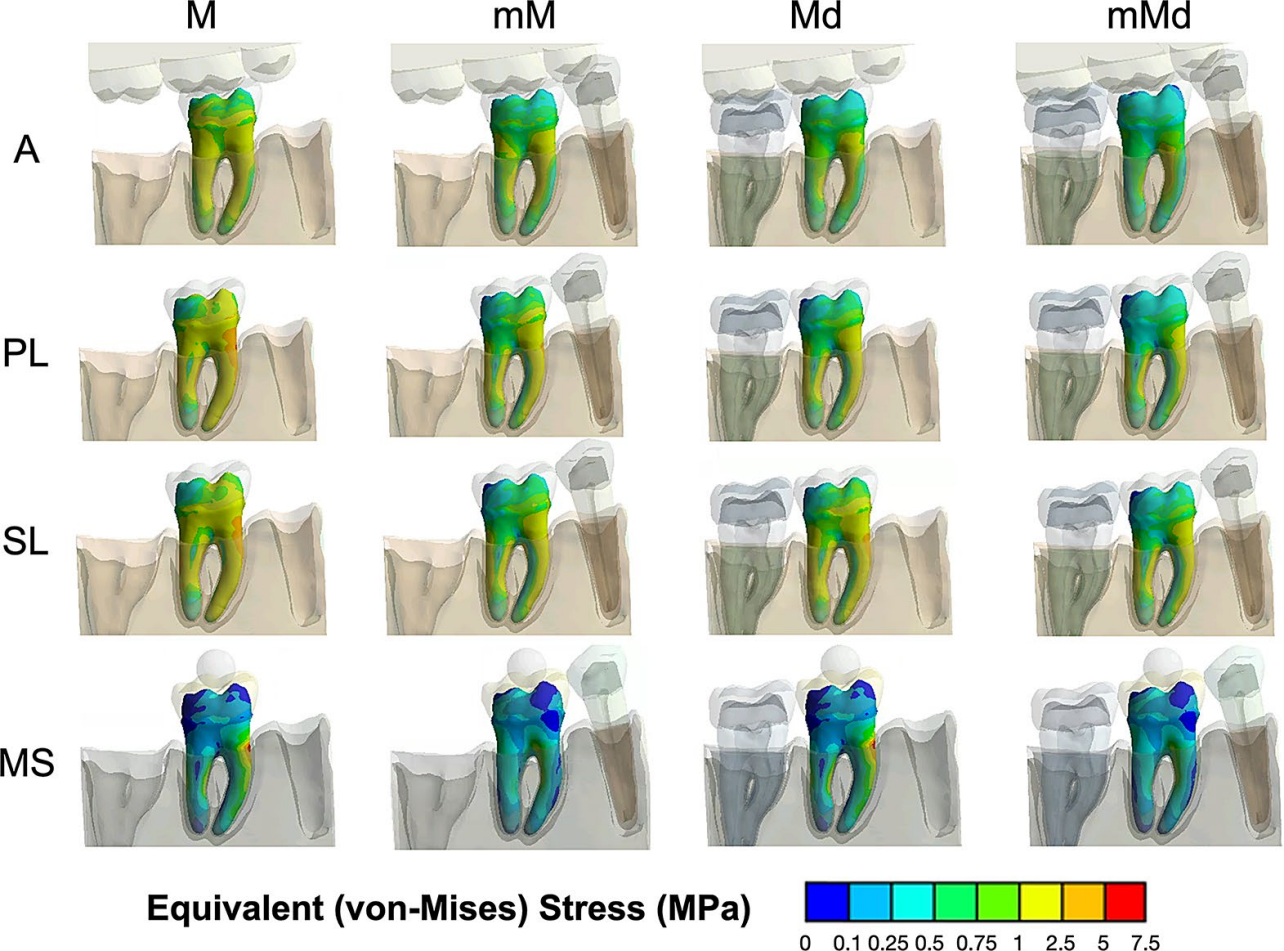
VM stress distributions in all FE models at 50N. High VM stresses are represented in red and low VM stresses in blue. Blue VM stresses imply areas that are not suffering relevant stresses in comparison to the red ones, in which the mechanical response is significant

Simulations were performed both with 50 N and 100 N loads, magnitudes that are within the reported range of functional biting loads exerted by healthy human adults [3, 17]. As shown in Fig. 2, the force was modelled to represent perfect antero-posterior occlusion (17.5º from the vertical axis) with no horizontal component [3, 41].

A fixed support boundary condition was applied to both anterior and posterior ends of cortical and trabecular bone, allowing no displacements in any of the limiting nodes of the model in accordance to similar studies [42, 43]. Surface contacts enamel-dentin, dentin-PDL, PDL-bone were defined as bonded. Surface contacts enamel-enamel (both mandibular-maxillary and proximal contacts) [44, 45] and enamel-sphere [46] were defined as frictional with a friction coefficient of 0.3.

### Data analysis

Equivalent VM stress was calculated along the entire dentin and PDL of the mandibular first molar for all mandibular configurations and loading application methods. Maximum VM stress was extracted from the dentin and compare among the different mandibular configurations and loading application methods. Locations of highest VM stress were hence determined and compared among simulations. In addition, simulations with the highest and lowest dentinal VM stress were selected for further analysis. VM strain of the PDL, total deformation of dentin and PDL and VM stress distribution in the PDL and bone were calculated at 100 N.

## Results

Figure 3 shows VM stress distributions in dentin for all FE models at 50N. 

**Fig. 4:**
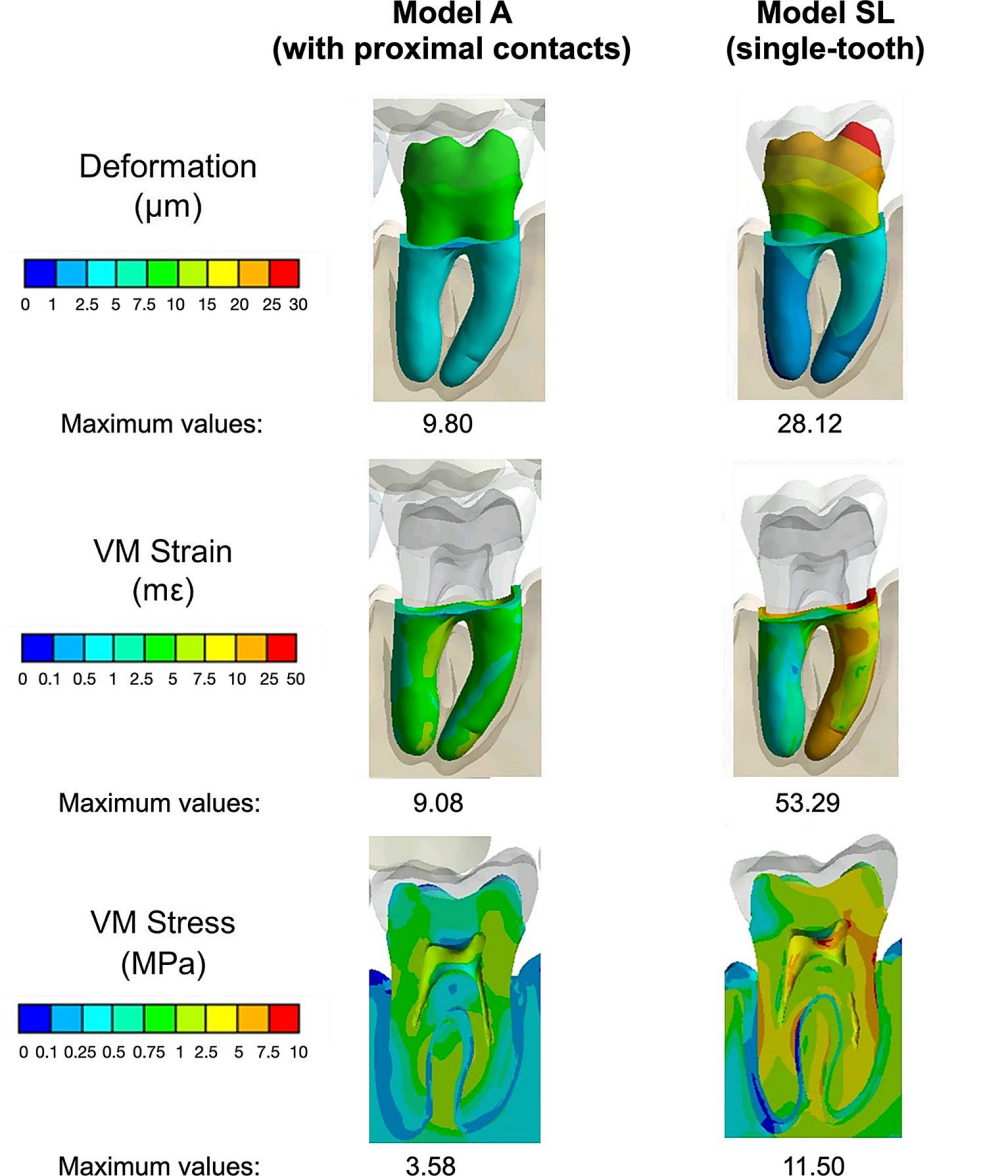
VM strain in the PDL, total deformation in PDL and dentin, and VM stress for dentin, PDL and bone for single-tooth SL model and the mandibular configuration with both proximal contacts for model A at 100N. Blue color represent low values for all three metrics, while red indicates higher values. The maximum values of each simulation and metric are also included

Table 2 presents the maximum VM stress values obtained for both 50 N and 100 N. For comparison the percentage of maximum VM stress for each mandibular configuration in relation to the single-tooth model (M) [MC/M], and the relative maximum VM stress ratio of SL, PL and MS models in relation to model A [LA/A] were also included.

**Table 2:** Maximum VM stress values, percentage of maximum VM stress for each mandibular configuration in relation to M [MC/M], and relative maximum VM stress ratio of SL, PL and MS models in relation to model A [LA/A] for both 50 N and 100 N

		Mandibular Configuration (MC)											
		M			mM			Md			mMd		
**Loading Applications (LA)**	Load (N)	Max VM (MPa)	% Max VM [MC/M]	ratio Max VM [LA/A]	Max VM (MPa)	% Max VM [MC/M]	ratio Max VM [LA/A]	Max VM (MPa)	% Max VM [MC/M]	ratio Max VM [LA/A]	Max VM (MPa)	% Max VM [MC/M]	ratio Max VM [LA/A]
**A**	50	4.01	N/A	N/A	2.76	68.83	N/A	2.62	65.34	N/A	2.08	51.87	N/A
	100	7.48			4.85	64.84		4.70	62.83		3.58	47.86	
PL	50	5.46		1.36	3.47	63.55	1.26	3.29	60.26	1.26	2.42	44.32	1.16
	100	10.92		1.46	7.00	64.10	1.44	5.97	54.67	1.27	4.30	39.38	1.20
SL	50	5.78		1.44	3.59	62.11	1.30	4.66	80.62	1.78	3.14	54.33	1.51
	100	11.50		1.54	7.28	63.30	1.50	9.26	80.52	1.97	6.13	53.30	1.71
MS	50	5.61		1.40	3.10	55.26	1.12	5.61	100.00	2.14	2.99	53.30	1.44
	100	11.08		1.48	6.27	56.59	1.29	11.08	100.00	2.36	6.08	54.87	1.70

Figure 4 displays VM strain in the PDL, total deformation in PDL and dentin and VM stress for dentin, PDL and bone for single-tooth SL model and the mandibular configuration with both proximal contacts for model A at 100 N.

In general, for all mandibular configurations and loading applications, the highest VM stress areas were consistently located in the cervical area of the mesial root and decreased in apical direction. VM stresses in the PDL were lower than those of its surrounding tissues (alveolar bone and dentin). Stress distributions differed among the loading applications and the presence of adjacent teeth reduced VM stress in dentin and PDL differently.

The highest VM stresses were found in the mesial root and decreased in the presence of adjacent teeth. The FE model with load application using a rigid metal sphere (MS) was the least influenced by the presence of adjacent teeth, whereas the stress distributions in the A model benefitted the most from their presence. Distal and mesial contacts influenced the distribution pattern differently for each loading application. In comparison to single-tooth configurations, VM stress was lower when one or two adjacent teeth were present (ranging 31–45% for mM, 0–45% for Md and 45–60% for mMd). While the presence of mesial contacts reduced VM stress in all the models, the presence of the distal adjacent tooth showed lower VM stress mainly in models A and PL.

Simulations with the highest and lowest VM stress were respectively the single-tooth SL model and the mandibular configuration with both proximal contacts for model A. As described before further analysis of these 2 models was accomplished for a detailed description of the surrounding tissues. Figure 4 displays VM strain in the PDL, total deformation in PDL and dentin and VM stress for dentin, PDL and bone for both simulations at 100 N. The analysis showed that dentin experiences the highest VM stress values, exceeding those in other tissues by over 50%. The highest stress outside the dentin was observed in the trabecular bone at the furcation area. At the same time, the highest VM strain was located in the cervical area of the mesial root for both simulations. However, as shown in Fig. 4, the model A with proximal contacts showed a very low maximum VM strain when compared to the SL single-tooth model. In terms of deformation, model A with proximal contacts showed higher deformations in the coronal portion of the dentin and lower deformation in the PDL, but all values were lower than 10 μm. Conversely, SL-M presented higher deformations. Maximum deformation occurred in the coronal third of the mesio-buccal cusp (28.12 μm) and decreased in an apical and distal direction.

Specifically, the pattern of stress distribution is described below for each loading application and mandibular configuration.

### Model A

This model was characterized by fewer areas with high VM stresses compared to the other 3 loading applications. As shown in Table 2, the model A showed lower VM stress (average = 2.87 MPa at 50 N and 5.15 MPa at 100 N) than PL (average = 3.66 MPa at 50 N and 7.05 MPa at 100 N), SD (average = 4.29 MPa at 50 N and 8.54 MPa at 100 N) and MS (average = 4.33 MPa at 50 N and 8.63 MPa at 100 N) models. In all mandibular configurations, higher VM stress concentrations were found in the cervical area of the mesial root and were reduced with the presence of any of the adjacent teeth.

### Model PL

This model showed the closest distributions of VM stress in both dentin and PDL to those found when using antagonist teeth for all mandibular configurations. As shown in Table 2, the relative ratio ranged from 1.16 to 1.46 in comparison to model A. The single-tooth mandibular configuration was the least favorable in terms of VM stress distribution.

### Model SL

While the SL model presented similar VM stress distributions to the model A, higher VM stresses were found in comparison to the PL model. As shown in Table 2, relative ratio ranged from 1.30 to 1.97 in comparison to model A; however, the presence of mesial proximal contacts reduced VM stress more than the presence of a distal adjacent tooth. M mandibular configuration showed the highest VM stress.

### Model MS

This model presents the highest VM stresses (Table 2). As shown in Table 2, relative ratio ranged from 1.12 to 2.36 in comparison to A models. It showed the highest VM stresses in the cervical area of the mesial root, although, the stresses were lower in the presence of a mesial proximal contact.

## Discussion

This study aimed to determine the differences in load distribution observable in a first mandibular molar with a FE analysis. This dental biomechanical model utilized functional occlusal load applications with antagonist teeth and other commonly used loading applications. Further, the study analyzed the changes in load distribution patterns in the presence of a single-tooth and when one or two adjacent contacts were present.

In most previous studies, FE models were often constructed using scans of extracted teeth [13, 47–50]. In contrast, the methodology of the present study sought to minimize simplifications inherent to this reductionist approach by using a high-resolution CBCT scan of both jaws from an actual patient.

This method appears to be useful for several reasons. First, the model provided a more detailed representation of all dental structures involved in occlusion, including adjacent and antagonist teeth, and hence allowed a more reliable reproduction of a clinical biomechanical behavior. Another advantage was, that similarly to some prior studies [47, 49], all relevant tissues were segmented and modelled: enamel, dentin, PDL, cortical and cancellous bone. In contrast, other previous studies have tended to simplify the reproduction of dental tissues, for example, by not differentiating cortical and cancellous bone [48–52] or disregarding the presence of enamel [53].

Unlike previous studies, this research aimed to investigate the role of adjacent teeth in dental biomechanics by modelling four different mandibular configurations. The CBCT scan included the first mandibular molar and both the second mandibular molar and premolar. In the most complete mandibular configuration (mMd), both adjacent teeth were preserved, and natural occlusal contacts were accurately reproduced. Additionally, other mandibular configurations were also analyzed by removing either the mesial (mM) or distal adjacent tooth (Md). A single-tooth model (M) was also modelled by removing both adjacent teeth, representing the classic model commonly used to study stress distribution patterns in dental biomechanics [47–50]. The absence of adjacent teeth in classic single-tooth models does not consider the dissipation of stress through the mechanical interaction of proximal contacts. As a result, the outcomes reported in previous studies may have been more pessimistic than what would be expected in clinical situations.

Furthermore, to assess the reliability of simple biomechanical models, the loading application with antagonist teeth was compared to other commonly used load applications in previous studies (point load in occlusal contact centroids [27, 28], distributed surface load in occlusal contact areas [26, 39] and rigid metal sphere in the mandibular first molar [38, 40]). In some studies, applied loads mimicked occlusal contacts by analyzing wear facets or using negative counterparts [48, 51]. Other prior work applied simulated loads in functional cusps and central fossa [47, 49] and yet further studies reproduced the occlusal contacts of a patient that were registered before tooth extraction [50]. Similarly, in the present study the masticatory load distribution was registered with an electronic pressure sensor and later reproduced in the model.

At the same time, occlusal loads are a compound of forces in different directions [3]. Reproducing the complex functional motions virtually is challenging, which is why various loading scenarios have been used in different studies, ranging from vertical loads [48] to angulated loads [47] or a combination of both angulated and vertical loads [49, 52]. In the present study, the load was applied simulating the antero-posterior occlusion, with a 17.5º angulation [41] from the vertical axis without any horizontal component. In the A loading application model, an accurate structural interaction of the dental complex was intended. The occlusal surface of the antagonist teeth was modeled from the enamel obtained in the CBCT scan of the maxilla, in an effort to replicate the relative motion between the maxillary and mandibular teeth, as well as the frictional interaction of both enamel surfaces during occlusal loads.

A clinically relevant finding in the current study was that the highest VM stress surface was consistently located at the cervical area of the mesial root and decreased in apical direction no matter the mandibular configuration or loading application. These results are in alignment with the usual failure mechanism of teeth and are also in accordance with previous FE studies [23, 25, 26, 46, 54, 55]. In fact, a high percentage of non-traumatic fractures are generated in the cervical area of the tooth [56, 57].

Another interesting result was that the presence of adjacent teeth reduced both VM stress in dentin and PDL differently. In fact, stress distributions differed among the loading applications and mandibular configurations. In general, the presence of both adjacent teeth was the most structurally favorable mandibular configuration, and the single-tooth model represented the least favorable dental biomechanical model. The presence of proximal contacts allows the transmission of the occlusal load, and the distribution of the stresses generated in the mandibular first molar towards the adjacent teeth. In line with previous findings [58], the presence of one or both proximal contacts influenced VM stress along the entire dentin and PDL complex; however, the relevance of the mesial or distal contact changed for the different loading applications. Although the influence of the distal adjacent tooth was more pronounced in decreasing VM stress in dentin for A loading application, the significance of the mesial proximal contact was more notable in SL and MS simulations. In PL simulations, the presence of a proximal contact, whether mesial or distal, did not exhibit substantial differences. This difference might be attributed to the displacement generated by the load application. The frictional occlusal contacts between mandibular and maxillary teeth in model A might generate a relative displacement between mandibular and maxillary crowns that also represents a more realistic movement of the dental complex. This relative displacement might displace the contact itself to a different position, changing the exact direction of the applied forces due to the normal contact between cusps. On the other hand, the displacement generated in models SL and MS pushed the first mandibular molar to the mesial contact area, and the point load of PL seemed not to significantly displace the tooth towards its proximal contacts.

At the same time, SL presented higher average von Mises stresses in both dentin and PDL. This finding was not expected due to the more distributed applied load closer to a clinical situation [26]; however, differences in the results obtained are relevant with 1.30 to 1.97 higher maximum VM stress ratio than functional models with antagonist teeth. In contrast, load application in model MS differs considerably from physiological occlusion due to the contact of a rigid sphere with the occlusal surface. Substantial differences were observed with this model. In accordance with previous studies [59], high cervical VM stresses were detected in the absence of a mesial proximal contact. It seems that when the sphere is indenting the enamel, it compresses the occlusal surface, producing the deformation of the surrounding surface and increasing the contact with the sphere generating tensile stresses in the external surfaces. This, in addition to the reaction force of the proximal contacts (which is above the center of mass of the first mandibular molar), generates a flexural motion which increases the stress in the buccal surfaces. Other studies also presented results with similar distributions [38, 60].

The present study shows that A models obtained the lowest VM stress out of the four analyzed loading applications. When the relative maximum VM stress ratio of simulated models in relation to A models were calculated, the closest results for each mandibular configuration were 1.36 for M, 1.12 for mM, 1.26 for Md and 1.16 for mMd mandibular configuration respectively for PL, MS and PL loading applications at 50 N. Results were similar for 100 N loads with slightly different values (1.46 for M, 1.29 for mM, 1.27 for Md and 1.20 for mMd). Likewise, M and mMd mandibular configurations obtained respectively the highest and lowest VM stresses for all loading applications. When the percentage of maximum VM stress for mMd mandibular configuration in relation to the single-tooth model (M) was calculated, an average reduction of 50.13%, 58.15%, 46.19 and 45.91% was detected respectively for A, PL, SL and MS load applications. These calculations demonstrated that the model with both proximal contacts and antagonist teeth showed the most conservative structural response.

Therefore, the results of the present study suggest that the use of different loading applications produces substantial differences in the structural response of dental tissues. Relevant differences in the stress distributions exerted along the dental complex were detected between PL, SL and MS and loads with antagonist teeth. However, the present results should be interpreted with some caution due to inherent limitations of FEA studies like the lack of experimental validation and the reliability of complex computer models of biological structures. When defining boundary conditions, the embeddings could have influenced the mechanical response differently across various mandibular configurations. Although previous studies similarly constrained the displacements of the boundary nodes [42, 43], such constraints might be mitigated in single-tooth models but affect the accuracy of deformation in the presence of proximal contacts, although the embeddings are far away from the mandibular molar analyzed in all simulations.

To avoid misinterpretation of the structural response of the dental tissues, close-to-reality boundary conditions should be chosen when analyzing dental biomechanical models. Future studies should assess the relevance of additional variables that could influence the structural response of teeth to find an accurate universal dental biomechanical model that balances the computational and/or manufacturing cost and the accuracy of the results. Further studies should also analyze the effect of these variables under cyclic loads. Future validation studies should also be performed evaluating other structural variables.

## Conclusions

Within the limitations of the present study, it can be concluded that:

- for all mandibular configurations and loading applications the cervical area of the mesial root experiences the highest stress that decreases in apical direction.
- both the presence of adjacent teeth and the loading applications influence the biomechanical behavior of teeth: the presence of proximal contacts and a masticatory load distribution with antagonist teeth shows the most conservative structural response.
- the presence of one or both proximal contacts reduces the stresses throughout the dental tissues respectively up to 44.7% or 60.6%. Single-tooth models represent the worst possible scenario and should only be extrapolated to patients with no adjacent teeth.
- loading applications that differ from the antagonist teeth may result in inaccurate stress distribution patterns and structural responses of the dental complex.

## Data Availability

No datasets were generated or analysed during the current study.
