# Correction to: Structural response of mandibular first molars in the presence of proximal contacts: finite element analysis with antagonist teeth and alternative loading applications

**DOI:** 10.1007/s00784-025-06648-z

**Published:** 2025-11-12

**Authors:** Saúl Dorado, Jesús R. Jimenez-Octavio, Paula Riaza, Ove A. Peters, Ana Arias

**Affiliations:** 1 Department of Mechanical Engineering, Escuela Técnica Superior de Ingeniería ICAI, Universidad Pontificia Comillas, Madrid, Spain; 2 MOBIOS Lab, Institute for Research in Technology, Escuela Técnica Superior de Ingeniería ICAI, Universidad Pontificia Comillas, Madrid, Spain; 3 Department of Conservative and Prosthetic Dentistry, School of Dentistry, Complutense University, Plaza Ramon y Cajal s/n. Ciudad Universitaria, Madrid, 28040 Spain; 4 Oral Health Centre, School of Dentistry, The University of Queensland, Herston, QLD Australia

**Correction to: Clinical Oral Investigations**

10.1007/s00784-025-06290-9

The article “Structural response of mandibular first molars in the presence of proximal contacts: finite element analysis with antagonist teeth and alternative loading applications”, written by Saúl Dorado, Jesús R. Jimenez-Octavio, Paula Riaza, Ove A. Peters and Ana Arias, was originally published Online First without Open Access. After publication in volume 29, issue 4, pages 469–480 the author decided to opt for Open Choice and to make the article an Open Access publication. Therefore, the copyright of the article has been changed to © The Author(s) 2025 and the article is forthwith distributed under the terms of the Creative Commons Attribution 4.0 International License, which permits use, sharing, adaptation, distribution and reproduction in any medium or format, as long as you give appropriate credit to the original author(s) and the source, provide a link to the Creative Commons licence, and indicate if changes were made. The images or other third party material in this article are included in the article’s Creative Commons licence, unless indicated otherwise in a credit line to the material. If material is not included in the article’s Creative Commons licence and your intended use is not permitted by statutory regulation or exceeds the permitted use, you will need to obtain permission directly from the copyright holder. To view a copy of this licence, visit http://creativecommons.org/licenses/by/4.0.

The original article has been corrected.

